# Supplementary material for: Selective on-surface covalent coupling based on metal-organic coordination template
Source: Nat Commun. 2019 Jan 8;10:70. doi: 10.1038/s41467-018-07933-0 (PMC6325127; doi:10.1038/s41467-018-07933-0)
Supplement: Supplementary file 1 — Supplementary Information [file 41467_2018_7933_MOESM1_ESM.pdf]

Supplementary Information for

Xing et al., **Selective On-Surface Covalent Coupling Based on Metal-Organic Coordination Template**

Supplementary Information for

Selective On-Surface Covalent Coupling Based on Metal-Organic Coordination Template

Shuaipeng Xing,[1] Zhe Zhang,[2] Xiyu Fei,[3] Wei Zhao,[4] Ran Zhang,[5] Tao Lin,[6] Danli Zhao,[3] Huanxin Ju,[7] Hu Xu,[2*] Jian Fan,[3*] Junfa Zhu,[7] Yu-qiang Ma[1,8*] and Ziliang Shi[1*]

[1]Center for Soft Condensed Matter Physics and Interdisciplinary Research & School of Physical Science and Technology, Soochow University, Suzhou 215006, China; [2]Department of Physics, Southern University of Science and Technology, Shenzhen 518055, China; [3]Institute of Functional Nano & Soft Materials (FUNSOM), Soochow University, Suzhou 215123, China; [4]Institute for Advanced Study, Shenzhen University, Shenzhen 518060, China; [5]Department of Physics, The Hong Kong University of Science and Technology, Clear Water Bay, Hong Kong; [6]College of New Materials and New Energies, Shenzhen Technology University, Shenzhen 518118, China; [7]National Synchrotron Radiation Laboratory, University of Science and Technology of China, Hefei 230029, China; [8]National Laboratory of Solid State Microstructures and Department of Physics, Nanjing University, Nanjing 210093, China

Supplementary Information

Supplementary Note 1. Details of the Br···Br interacted molecular chain structure.

Supplementary Note 2. Unstable assembly of the *p*-**DBTB** in the presence of Fe at room temperature.

Supplementary Note 3. XPS measurements.

Supplementary Note 4. DFT calculations of the organometallic L-mode links.

Supplementary Note 5. Band structure of organic SPy-*p*-Ph ribbons.

Supplementary Note 6. DFT calculations of the II- and III-mode SPy units.

Supplementary Note 7. Inhomogeneous polymorphic structures of *m*-**DBTB.**

Supplementary References

Supplementary Note 1. Details of the Br···Br interacted molecular chain structure.

To investigate the Br···Br interactions in the chain structure (see Figure 4 in the main text), the Br···Br distances and the CBr···Br angles of the structural model of the chain were analysed. The geometry of the *cis*-D_{2h} conformers, with one Fe atom coordinating with the Br-tpy terminal, is obtained by fully relaxing the atomic positions of the monomers in a 32×32×21 Å vacuum region. Halogen bonding (X-bonding) can be classified into two types based on the CX···X angles[1,2], namely type-I bond (repulsive) and type-II (attractive). In the structural model (Supplementary Figure 1), both Br1···Br2 and Br3···Br4 bonding motifs are identical. Each possesses an unsymmetrical configuration, with the angles of 165°and 127° (see the table in Supplementary Figure 1). This configuration suggests an attractive type-II bonding. The Br2···Br3 interaction has a symmetrical configuration with the angle of 156° (for both CBr2Br3 and CBr3Br2), and thus can be assigned to a repulsive type-I bonding. The distances of these Br-Br bonds lie in the typical range of non-covalent halogen bonds[1-3]. Thus, a net attractive interaction between two molecular units is feasible, which is responsible for the formation of the chains.

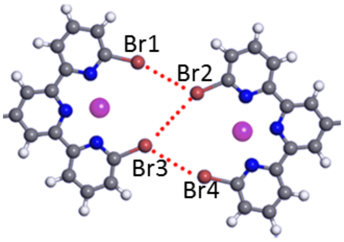

Distance/Å		Angle/°			
Br1···Br2	3.8	CBr1Br2	165	CBr2Br1	127
Br2···Br3	4.2	CBr2Br3	156	CBr3Br2	156
Br3···Br4	3.8	CBr3Br4	127	CBr4Br3	165

Supplementary Figure 1| The close-up look of the structural model (left) and the derived distances and angles (right) of the Br···Br bonding motifs between the two adjacent Br-tpy terminals.

Supplementary Note 2. Unstable assembly of the *p*-DBTB in the presence of Fe at room temperature.

After the codeposition of Fe and the *p*-**DBTB** molecules on Au(111), STM monitoring of the sample held at room temperature (293 K) resolved a few molecular clusters (Supplementary Figure 2). These clusters were probably stabilized by intermolecular Br-Br halogen bonds or immobilized by Fe clusters, while most molecules existed in a lattice-gas phase and thus were invisible. Indeed, the molecular clusters were unstable and subject to reorganize during scanning, as indicated by the dashed circles. Therefore, the preorganization of the *p*-**DBTB** chain structure (at *T*=100 K) is unlikely responsible for guiding the next C-C coupling reaction.

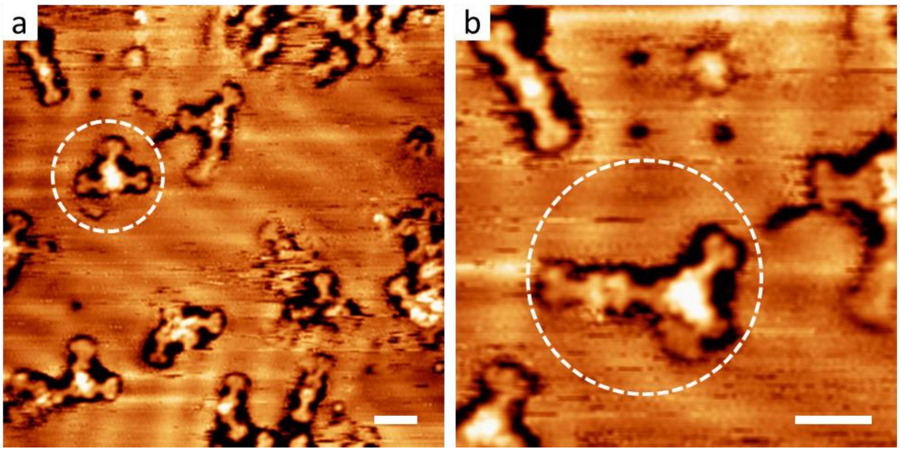

Supplementary Figure 2 | STM observation of the sample coadsorbed with Fe and molecules at room temperature (293 K). (a) STM overview. (b) The close-up inspection of the *p*-D**BTB** clusters. Circles indicate that a molecular cluster changes its arrangement during the scanning. Scale bars: 3 nm.

Supplementary Note 3. XPS measurements.

To examine the reaction pathway of the molecules in the presence of Fe, complementary high-resolution XPS experiments were conducted regarding the organometallic chain phase and the conjugated SPy-*p*-Ph ribbon phase. The evolution of Br 3d, C 1s spectra (Supplementary Figures 3a,b) was collected after the annealing at 423 and 603 K, respectively. The corresponding reaction pathway is illustrated in Supplementary Figure 3c; for clarity, the Fe coordination atoms are not shown. As shown in the upper panel of Supplementary Figure 3a, Br $3d_{5/2}$ and $3d_{3/2}$ are clearly resolved with the binding energy at 68.0 and 69.0 eV, respectively. The two split doublets ($3d_{5/2}$ and $3d_{3/2}$) differ by 1.0 eV, and the intensity ratio is exactly 3:2, in line with the features of Br 3d in XPS Handbook. This spectrum identifies exclusively one chemical environment for Br adatoms, indicating only one Br species on the surface. According to the literatures[4-7], the Br species can be assigned to the Br atoms chemisorbed on the Au(111) surface. This observation agrees with our STM results of the organometallic chain phase, where the cleaved Br atoms were populated in the inter-chain spaces. After annealing at 603 K, the Br 3d signal disappeared (Supplementary Figure 3a, bottom), indicating a complete Br desorption at this temperature, in agreement with the literatures[5-7].

The C 1s spectrum measured after the annealing at 423 K shows a major peak at 284.4 eV and a shoulder at 285.8 eV, respectively (Supplementary Figure 3b). The major peak can be attributed mainly to the C atoms from C-H. The shoulder can be ascribed to the C atoms from py and bonding with the N atom[5,8], which have two chemical states by further bonding with Au or C (labelled 1 or 2; see the reaction mode in Supplementary Figure 3c). The whole C 1s spectrum shifts to a higher binding energy by ~0.4 eV from 423 K to 603 K, presumably due to the Br desorption and the C-Au-C cleavage followed by C-C coupling reaction[6,9]. The increment of the shoulder intensity can be attributed to the fact that the C-Au-C bonds convert to the C-C bonds; *i.e.*, the carbon 1 transforms into 2 (Supplementary Figure 3c). The results also agree with our STM observation, where the covalent SPy-*p*-Ph ribbons have emerged after the annealing at 578-705 K.

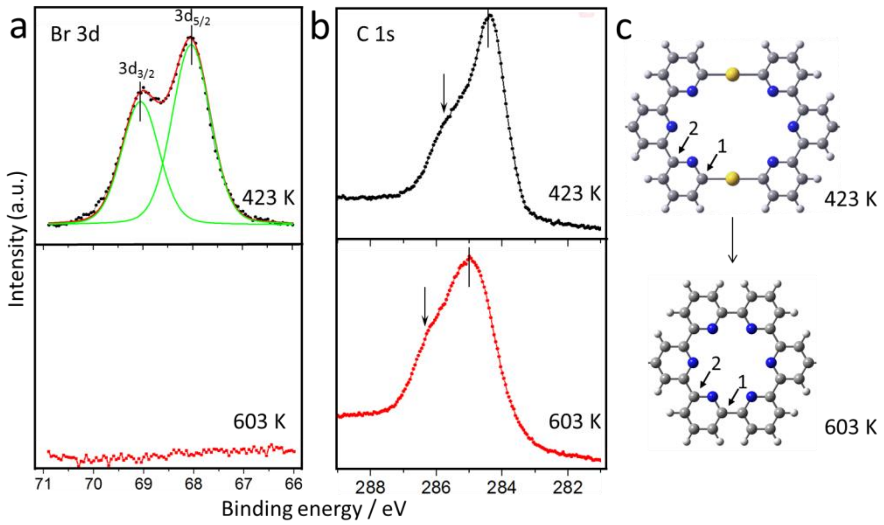

Supplementary Figure 3 | XP spectra (*hv*=380 eV) collected after annealing of *p*-**DBTB**/Au(111) in the presence of Fe at 423 K and 603 K. (a) Br 3d. The spectrum at 423 K is deconvoluted using XPS peak 41 program after subtraction of a Shirley background. (b) C 1s. The major peak and the shoulder are denoted by the bar and the arrow, respectively. (c) Reaction model. Two different carbon atoms are denoted with 1 and 2. During the annealing processes, carbon 1 converted to carbon 2, leading to an increment of the shoulder intensity in C 1s spectra.

Supplementary Note 4. DFT calculations of the organometallic L-mode links.

First, our STM measurements have shown that the Br/molecule ratio in the organometallic chain domains is 4. Considering that most of the L-mode links exhibit the similar bright feature at the center, and that most of Br atoms are seen stabilizing in between the chains, we may exclude the attribution of Br to the bright feature of the L-mode link. Furthermore, our complementary XPS measurements (Supplementary Figure 3a) show solely the Br atoms chemisorbed on Au(111) in the organometallic chain phase, in agreement with our STM observation.

Second, the Fe_3-tpy structural model (labelled Fe_3) similar to the reported Fe-coordination on Ag(111)[10,11] was excluded. The Fe_3 model was built by placing a linear Fe trinulcear cluster in between two C-Au-C connected molecular units. The DFT-optimized structure indicates that the three Fe atoms sink downwards with respect to both the molecular plane and the Au atoms (within the C-Au-C bonds) (Supplementary Figure 4a). The simulated STM topographs (U=-0.05 V, Supplementary Figure 4b; U=-0.5 V, Supplementary Figure 4c) show a relatively dim center, resembling to the Fe_3-tpy coordination on Ag(111), but distinct from our high-resolution STM observations.

Last, taking into account that both Au and Fe atoms exist on the surface, and can be captured by py ligands[12-15], we have conducted DFT calculations on the two models with either a Au (labelled Fe_3-Au) or an Fe atom (labelled Fe_3-Fe) beneath the central Fe atom of an Fe_3-tpy coordination. The DFT-optimized Fe_3-Au model shows a stable configuration where the central Fe atom is pushed up due to the extra Au adatom beneath. The molecule-substrate distance is about 3.2 Å. However, in the Fe_3-Fe model the underneath Fe atom tends to dive into the gold substrate, leaving an Fe_3 cluster (Supplementary Figure 4d) similar to the Fe_3-model. Thus, the Fe_3-Fe model can be excluded. The validity of the Fe_3-Au model was further corroborated by the STM simulations with the bias voltage U=-0.05 V. In contrast with the Fe_3 model, the Fe_3-Au model reproduces the bright feature for the central Fe of the L-mode link; see Supplementary Figures 4c, e and f.

On the basis of these theoretical analysis, we propose that the lifting of the central Fe atom, due to its stacking configuration with the Au adatom underneath, attributes to the bright feature observed in STM topographs. Distinct from the Fe_3-tpy coordination on Ag(111), the

Au-adatom is incorporated into the Fe-coordination on Au(111), which is likely due to the aurophilicity of the gold[16]. Unambiguously determining the state of the central Fe atom needs state-of-the-art techniques, including tip-manipulation and scanning tunneling spectroscopy working at cryogenic temperatures[10,11], and thus has not been done with our instrument.

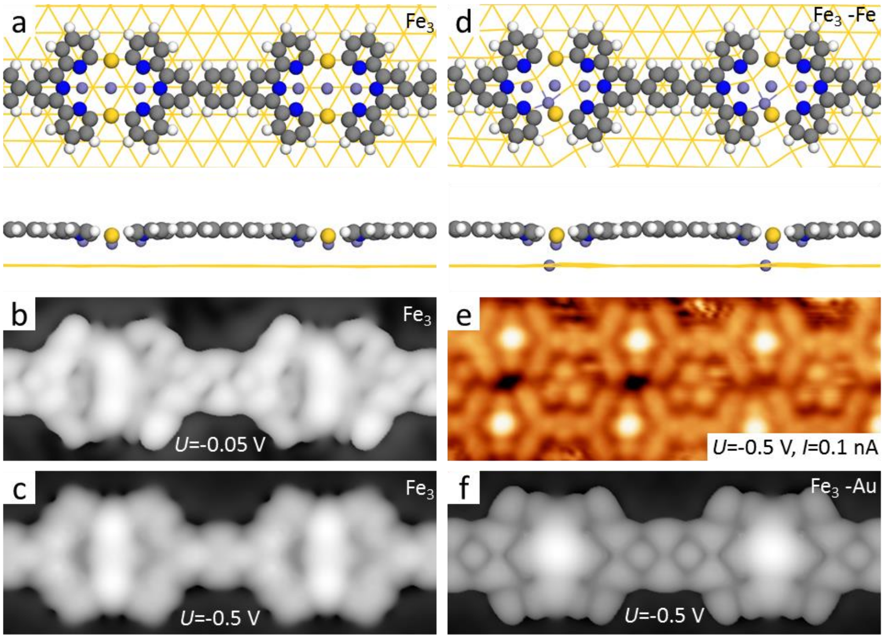

Supplementary Figure 4 | DFT calculations of the organometallic L-mode links. (a-c) Top view, side view and STM simulations of the DFT-optimized Fe_3 model. C, grey; N, blue; H, white; Au adatom, yellow; Fe, purple. (d) Top view and side view of the DFT-optimized Fe_3-Fe model. (e) High-resolution STM image of the organometallic chains with tunneling parameters *U*=-0.5 V, *I*=0.1 nA. (f) DFT simulation (*U*=-0.5 V) of the Fe_3-Au model.

Supplementary Note 5. Band structure of organic SPy-*p*-Ph ribbons.

Electronic band structure and density of states of SPy-*p*-Ph nanoribbons are shown in Supplementary Figure 5. The spatial structure of charge density of conduction band minimum (CBM) and valence band maximum (VBM) are visualized using an isosurface of 0.005 *e* $Bohr^{-3}$.

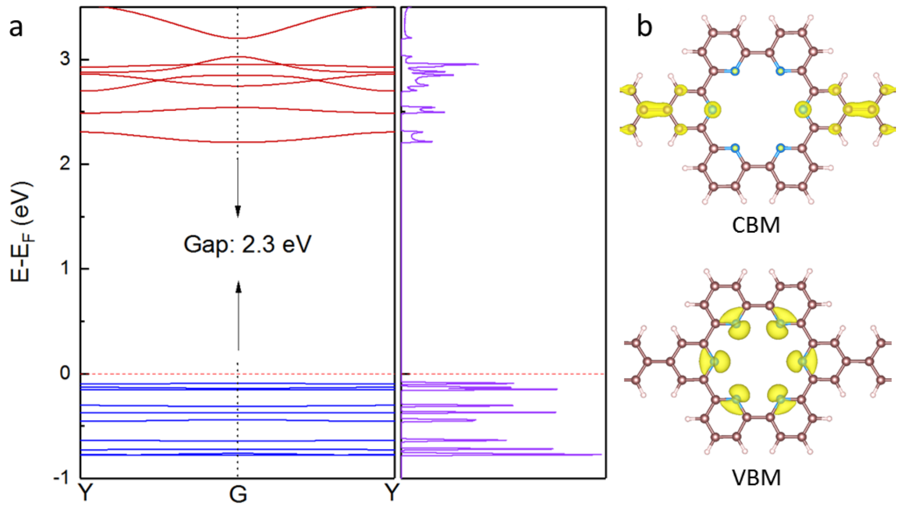

Supplementary Figure 5 | (a) Electronic band structure and density of states of the SPy-*p*-Ph nanoribbon. The blue and red lines correspond to occupied and unoccupied states, respectively. (b) Spatial distribution of CBM and VBM. Colors: N, blue; C, brown; H, white; charge density, yellow.

Supplementary Note 6. DFT calculations of the II- and III-mode SPy units.

Our complementary XPS measurements revealed that the Br atoms desorbed completely after annealing at 603 K (Supplementary Figure 3a). As revealed in the STM observation, the abundance of II/III-mode SPys was much higher than the I-mode after annealing at 650 K; see Figure 6f in the main text. Thus, we may exclude the binding of Br atoms to the II/III-mode SPy units. The structural models of the II- and III-mode SPy units were established with the guest species of a single Fe atom (II-mode; Supplementary Figures 6a-c), or an $FeAu_2$ cluster (1 Fe atom with 2 Au atoms underneath, III-mode; Supplementary Figures 6d-f). The STM simulation of the II-mode shows a moderately bright center, in good agreement with the corresponding STM topograph (Figure 6d in the main text). The $FeAu_2$ cluster in III-mode manifests a stable stacking configuration, and the STM simulation (U=-0.5 V) renders a much bright center, agreeing qualitatively with the STM result (Figure 6e in the main text). Considering that the III-mode SPy units emerged just after the disappearance of the C-Au-C organometallic phase during our annealing processes, we postulate that the two Au atoms are residuals out of the deformation of organogold L-mode links.

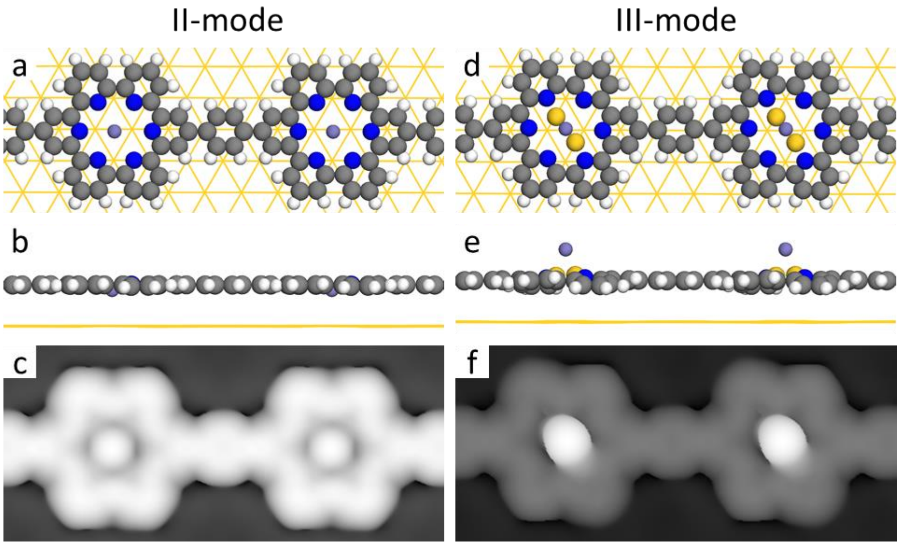

Supplementary Figure 6 | DFT-optimized structures and STM simulations of the II- and III-mode SPy units. (a-c) Top view, side view and STM simulation (U=-1.5 V) of the II-mode SPy. (d-f) Top view, side view and STM simulation (U=-0.5 V) of the III-mode SPy.

Supplementary Note 7. Inhomogeneous polymorphic structures of *m*-DBTB.

After depositing the compound 1,3-bis(6,6"-dibromo-[2,2':6',2"-terpyridin]-4'-yl)benzene (*m*-**DBTB**, see the inset of Supplementary Figure 7a) on a pristine Au(111) surface held at 293 K, the annealing treatment at 577 K was performed to initiate the coupling reaction between molecules. STM overview reveals an inhomogeneous polymorphic structure (Supplementary Figure 7a). Close inspections manifest three typical sub-structures, including nearly-triangular pores (Supplementary Figures 7b,c) and irregular polygons (Supplementary Figure 7d). Based on our structural analysis, the tentative structural models (lower panels in Supplementary Figures 7b-d) are proposed to illustrate the coupling modes in these structures. It suggests that in the polymorphic structures, the molecules are covalently coupled through their tpy terminals, while most of bpy fragments appear in the *trans*-conformation. The formation of the nearly-triangular pores could be induced by the cyclodehydrogenation or direct arylation reaction[17].

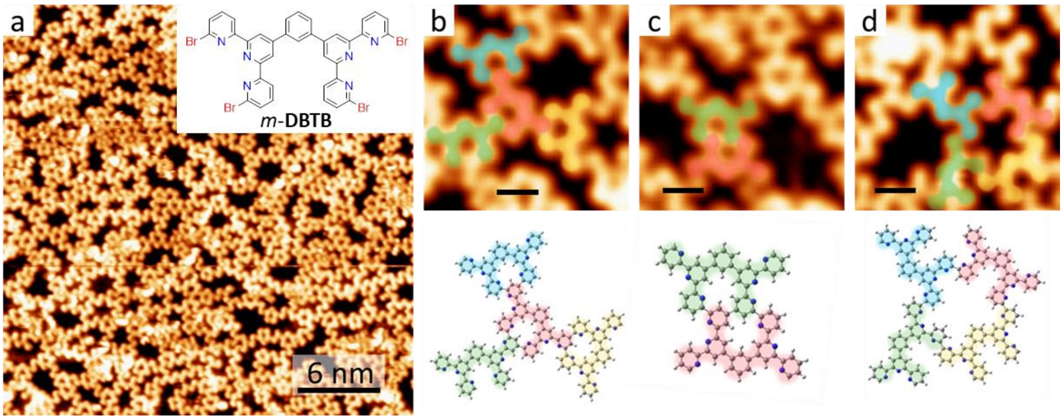

Supplementary Figure 7 | Inhomogeneous polymorphic structures of the *m*-**DBTB** molecules on Au(111). (a) STM overview. Inset: chemical structure of *m*-**DBTB**. (b-d) Three typical sub-structures and the tentative structural models. Molecular monomers are highlighted in different colors. Scale bars: 1 nm. Data acquisition conditions (T=293 K): (a,b,d) -0.8 V, 1.0 nA; (c) -0.8 V, 2.0 nA.

Supplementary References

1 Pham, T. A., Song, F., Nguyen, M.-T. & Stöhr, M. Self-assembly of pyrene derivatives on Au(111): substituent effects on intermolecular interactions. *Chem. Commun.* **50,** 14089-14092 (2014).

2 Bui, T. T. T., Dahaoui, S., Lecomte, C., Desiraju, G. R. & Espinosa, E. The Nature of Halogen⋯Halogen Interactions: A Model Derived from Experimental Charge-Density Analysis. *Angew. Chem. Int. Ed.* **48**, 3838-3841 (2009).

3 Lackinger, M. Surface-assisted Ullmann coupling. *Chem. Commun*. **53**, 7872-7885 (2017).

4 Basagni, A., *et al.*, On-Surface Photo-Dissociation of C-Br Bonds: Towards Room Temperature Ullmann Coupling. *Chem. Comm.* **51**, 12593-12596 (2015).

5 Smykalla, L., Shukrynau, P., Korb, M., Lang, H. & Hietschold, M. Surface-confined 2D polymerization of a brominated copper-tetraphenylporphyrin on Au(111). *Nanoscale* **7**, 4234-4241, (2015).

6 Batra, A. *et al.*, Probing the Mechanism for Graphene Nanoribbon Formation on Gold Surfaces through X-Ray Spectroscopy. *Chem. Sci*. **5**, 4419-4423 (2014).

7 Krasnikov, S. A. *et al.*, Formation of extended covalently bonded Ni porphyrin networks on the Au(111) surface. *Nano Res*. **4**, 376-384, (2011).

8 Yu, S. *et al.*, Modification of Charge Transfer and Energy Level Alignment at Organic/Tio2 Interfaces. *J. Phys. Chem. C*, **113**, 13765-13771 (2009).

9 Björk, J.; Hanke, F.; Stafström, S. Mechanisms of halogen-based covalent self-assembly on metal surfaces. *J. Am. Chem. Soc*. **135**, 5768-5775 (2013).

10 Krull, C. *et al.*, Iron-based trinuclear metal-organic nanostructures on a surface with local charge accumulation. *Nat. Commun.* **9**, 3211 (2018).

11 Schiffrin, A. *et al.*, Designing Optoelectronic Properties by On-Surface Synthesis: Formation and Electronic Structure of an Iron-Terpyridine Macromolecular Complex. *ACS Nano* **12**, 6545-6553 (2018).

12 Song, Y. *et al.*, Self-Assembly and Local Manipulation of Au-Pyridyl Coordination Networks on Metal Surfaces. *ChemPhysChem* **18**, 2088-2093 (2017).

13 Pawlak, R. *et al.*, Design and Characterization of an Electrically Powered Single Molecule on Gold. *ACS Nano* **11**, 9930−9940 (2017).

14 Shi, Z. *et al.*, Thermodynamics and selectivity of two-dimensional metallo-supramolecular self-assembly resolved at molecular scale. *J. Am. Chem. Soc*. **133**, 6150-6153 (2011).

15 Shi, Z. & Lin, N. Structural and Chemical Control in Assembly of Multicomponent Metal−Organic Coordination Networks on a Surface. *J. Am. Chem. Soc*. **132**, 10756-10761 (2010).

16 Zhang, H. & Chi, L. Gold–Organic Hybrids: On-Surface Synthesis and Perspectives. *Adv. Mater.* **28**, 10492-10498 (2016).

17 Alberico, D., Scott, M. E. & Lautens, M. Aryl−Aryl Bond Formation by Transition-Metal-Catalyzed Direct Arylation. *Chem. Rev.* **107**, 174-238 (2007).
